# Comparative effects of corticosteroid injection combined with mobilization vs. mobilization alone on pain, function, and radiological outcomes in university volleyball players with subacromial impingement syndrome: a prospective randomized controlled trial

**DOI:** 10.3389/fspor.2026.1834202

**Published:** 2026-09-03

**Authors:** Gopal Nambi, Mshari Alghadier, Osama R. Aldhafian, Alaa Jameel A. Albarakati, Shanmugananth Elayaperumal, Vijayamurugan Eswaramoorthi, Ramprasad Muthukrishnan

**Affiliations:** 1 Department of Health and Rehabilitation Sciences, College of Applied Medical Sciences, Prince Sattam bin Abdulaziz University, Al Kharj, Saudi Arabia; 2 Department of Surgery, College of Medicine, Prince Sattam bin Abdulaziz University, Al Kharj, Saudi Arabia; 3 Department of Surgery, College of Medicine, Al–Qunfudah Branch, Umm Al–Qura University, Makkah, Saudi Arabia; 4 Department of Physiotherapy, Sri BalajiVidyapeeth, Mahathma Gandhi Medical College & Rehabilitation Institute, Pondycherry, India; 5 Department of Physiotherapy, Faculty of Pharmacy and Health Sciences, Universiti Kuala Lumpur, Royal College of Medicine Perak, Ipoh, Malaysia; 6 Department of Physiotherapy, College of Health Sciences, Gulf Medical University, Ajman, United Arab Emirates

**Keywords:** corticosteroid, mobilization, randomized controlled trial, subacromial impingement syndrome, volleyball players

## Abstract

**Background:**

Subacromial impingement syndrome (SIS) is a common cause of shoulder pain and functional limitation in overhead athletes, particularly volleyball players. Although corticosteroid injections provide short-term pain relief and mobilization improves shoulder mechanics, evidence comparing their combined effect with mobilization alone in young athletic populations remains limited, particularly with respect to radiological outcomes.

**Objective:**

To compare the effects of corticosteroid injection combined with mobilization versus mobilization alone on pain, shoulder function, range of motion, and radiological outcomes in university volleyball players with SIS.

**Methods:**

This prospective randomized controlled trial included 60 male university volleyball players (18–25 years) with clinically diagnosed SIS. Participants were randomly allocated to receive either a single subacromial corticosteroid injection followed by a standardized mobilization program (*n* = 30) or mobilization alone (*n* = 30). Outcomes were assessed at baseline, 4 weeks, 8 weeks, and 6 months and included pain intensity (VAS, primary outcome), pain pressure threshold, shoulder range of motion, and functional disability (SPADI) as key secondary outcomes, and radiological measures (coracohumeral interval, supraspinatus tendon thickness, and subacromial bursa thickness) as exploratory outcomes. Intention-to-treat analysis and effect sizes were calculated.

**Results:**

Both groups demonstrated significant improvements over time; however, the combined intervention group showed significantly greater and earlier improvements in pain, function, and shoulder mobility (*p* = 0.001). Pain intensity (VAS) decreased significantly more in the combined intervention group, with a between-group mean difference of 2.6 points at 6 months (95% CI: 2.31 to 2.88), favoring the combined group. Pain pressure threshold increased significantly, with a between-group difference of 25.2 kPa at 6 months (95% CI: 8.6 to 41.8). Between-group differences exceeded minimal clinically important difference values for pain and SPADI, with moderate to large effect sizes. Radiological outcomes demonstrated greater increases in the coracohumeral interval and greater reductions in tendon and bursal thickness in the combined group at 6 months.

**Conclusion:**

Corticosteroid injection combined with mobilization provides superior and clinically meaningful improvements in pain, function, shoulder mobility, and radiological outcomes compared with mobilization alone in university volleyball players with SIS. This multimodal approach appears effective for managing SIS in high-demand overhead athletes. The imaging findings should be regarded as exploratory and supportive of improved subacromial mechanics rather than as evidence of definitive structural recovery.

**Clinical Trial Registration:**

identifier CTRI/2020/06/025692.

## Introduction

Subacromial impingement syndrome (SIS) is among the most prevalent shoulder disorders affecting athletes who regularly perform overhead activities. Volleyball players are particularly susceptible because of the repetitive, high-demand use of the shoulder complex required during the sport. The prevalence of SIS among overhead athletes varies depending on the sport, competitive level, and diagnostic criteria used (1). Previous studies indicate that shoulder impingement symptoms affect approximately 5%–36% of overhead athletes during their sporting careers (2). Volleyball requires repetitive overhead skills such as serving, spiking, blocking, and setting, involving forceful elevation, abduction, and rotation of the glenohumeral joint. These high-velocity movements generate considerable stress within the subacromial space, especially when combined with frequent jumping, rapid landings, and coordinated trunk and upper-extremity movements (3). Repeated exposure may result in cumulative microtrauma, inflammatory changes, tendon and bursal thickening, and progressive mechanical compression beneath the acromion, leading to shoulder pain and reduced range of motion (4). Because intensive training often begins during adolescence, structural adaptations such as tendon degeneration, bursal irritation, and altered scapular kinematics may accumulate over several years before symptoms become clinically evident (5).

Traditional management of SIS focuses on pain reduction and restoration of shoulder function through pharmacological and rehabilitation-based interventions. Corticosteroid injections are commonly administered for short-term pain relief but do not address the underlying biomechanical impairments associated with SIS (6). Mobilization- and exercise-based rehabilitation effectively improves pain and shoulder function; however, persistent pain may limit rehabilitation participation in overhead athletes (7). Therefore, combining corticosteroid injection with mobilization may improve early pain control and facilitate functional recovery compared with mobilization alone (8).

Integrated pharmacological and rehabilitation strategies are increasingly used in overhead athletes. Corticosteroid injections reduce subacromial inflammation and pain, whereas joint mobilization restores shoulder mobility, normalizes joint mechanics, and improves function (6, 9). Although mobilization-based rehabilitation is effective, pain often limits optimal participation among university volleyball players (7). Consequently, combining corticosteroid injection with mobilization may provide synergistic benefits by enabling more effective rehabilitation and improving both clinical and radiological outcomes (1). However, evidence comparing this combined approach with mobilization alone in young overhead athletes remains limited (8).

Most previous studies have evaluated corticosteroid injections or mobilization independently, with relatively few examining their combined effects in SIS rehabilitation (6, 9). In patients with subacromial pain syndrome, combining pharmacological treatment with supervised physiotherapy has demonstrated superior improvements in pain, shoulder range of motion, and functional performance compared with physiotherapy alone (8). These findings suggest that reducing inflammation and pain through corticosteroid injection may allow mobilization techniques to be performed more effectively within a pain-free range, thereby enhancing shoulder mobility, functional performance, and overall clinical outcomes. Although corticosteroid injections provide short-term analgesic and anti-inflammatory effects, the mechanisms underlying their interaction with mobilization remain incompletely understood. Early pain reduction may decrease nociceptive inhibition and muscle guarding, facilitating restoration of glenohumeral and scapulothoracic mechanics through manual therapy. Nevertheless, mechanistic evidence for this interaction, particularly in young athletic populations, remains limited.

Despite their widespread use, concerns exist regarding repeated or inappropriate corticosteroid injections, particularly in young athletes. Experimental and clinical evidence suggests that repeated corticosteroid administration may contribute to tendon matrix alterations, collagen disorganization, and potential cartilage damage. Although a single injection is generally considered safe, its long-term structural effects in high-demand athletic shoulders remain uncertain. Corticosteroid administration must also comply with current World Anti-Doping Agency (WADA) regulations, including route-specific restrictions and, when applicable, Therapeutic Use Exemptions (10). Therefore, both the potential benefits and risks should be carefully considered before corticosteroid use in young athletic populations.

Clinical and radiographic evaluations of combined corticosteroid injection and mobilization remain limited. Most previous investigations have focused on clinical outcomes, including pain intensity, range of motion, and functional disability, with relatively little assessment of structural changes (6, 9). Imaging parameters such as coracohumeral interval space, supraspinatus tendon thickness, and subacromial bursa thickness may provide valuable insight into biomechanical and tissue-level adaptations associated with treatment (11, 12). Assessing radiological alongside clinical outcomes may therefore clarify whether symptomatic improvement corresponds to measurable structural recovery, addressing an important gap in the current literature.

Although corticosteroid injection combined with structured rehabilitation is widely accepted in managing shoulder pain (7, 8), its effectiveness has primarily been investigated in general clinical or occupational populations using pain and disability outcomes alone. The present study extends existing evidence by evaluating a homogeneous cohort of competitive university volleyball players, a population characterized by unique biomechanical loading patterns and return-to-sport demands. Furthermore, it combines clinical outcomes with objective MRI and ultrasound measures of the coracohumeral interval, supraspinatus tendon, and subacromial bursa to determine whether symptomatic improvement is accompanied by measurable structural changes. Participants will also be followed for six months to evaluate medium-term, sport-relevant recovery. To our knowledge, this combination of athletic population, dual clinical-radiological assessment, and extended follow-up has not previously been reported for this intervention comparison.

Therefore, this prospective randomized controlled trial aims to compare the effectiveness of corticosteroid injection combined with mobilization versus mobilization alone on pain, shoulder function, and radiological outcomes in university volleyball players with subacromial impingement syndrome. We hypothesize that the combined intervention will produce superior improvements in pain relief, functional performance, and structural recovery, as assessed clinically and radiographically, compared with mobilization alone.

## Methods

### Study design

This is a prospective, randomized, parallel-arm controlled trial with a 4-week intervention phase and follow-up evaluation at 6 months, conducted per CONSORT 2022 recommendations. Ethical approval was obtained from the Departmental Ethical Committee of Prince Sattam bin Abdulaziz University, Al Kharj, Saudi Arabia (approval no. RHPT/020/026). Written informed consent was obtained from all participants, who were informed of their right to withdraw at any time without penalty; datasets were anonymized before analysis. The study complied with the Declaration of Helsinki (2013 revision) and was prospectively registered with the Clinical Trials Registry of India on 08 June 2020 (CTRI/2020/06/025692) prior to enrollment. Recruitment, initially planned for late 2020, was delayed by COVID−19-related restrictions, including suspension of university sports activities and limited access to clinical and imaging facilities; enrollment was subsequently conducted between May 2023 and April 2025. No amendments were made to the registered protocol.

Eligible participants were allocated 1:1 to the intervention group (corticosteroid injection combined with mobilization, *n* = 30) or the control group (mobilization alone, *n* = 30). Random sequence generation used Random Allocation Software (version 2.0), performed by an independent investigator uninvolved in recruitment, intervention delivery, or analysis. Allocation concealment was ensured using sequentially numbered, opaque, sealed envelopes prepared by a researcher not involved in enrollment or outcome assessment; group assignments were revealed only after baseline measurements were completed.

Because the interventions (corticosteroid injection versus mobilization alone) could not be concealed without a sham procedure, participant and therapist blinding was not feasible, risking performance bias and expectation-related effects in self-reported outcomes such as pain and disability. To minimize these risks, treatment protocols were standardized, with therapists instructed to maintain consistent communication, encouragement, and session structure across groups, and no additional therapeutic interventions were permitted. The study was therefore conducted as single-blinded, with outcome assessors and statistical analysts blinded to group allocation. Although the trial registry initially indicated participant blinding, the finalized protocol did not include it; the registry record will be updated to reflect the executed design.

### Risk of bias assessment

Risk of bias was assessed using the Cochrane Risk-of-Bias tool for randomized trials (RoB 2.0) across five domains. (1) Bias from the randomization process was low risk, given computer-generated allocation and concealment via sealed envelopes. (2) Bias from deviations from intended interventions was rated some concerns, as participants and therapists were unblinded and no sham injection was used, though standardized protocols were strictly enforced across both arms. (3) Bias from missing outcome data was low risk, given intention-to-treat analysis and minimal attrition (maximum two participants per group at any follow-up). (4) Bias in measurement of the outcome was low risk for objective, assessor-blinded outcomes (range of motion, pain pressure threshold, radiological measures) but some concerns for self-reported outcomes (VAS and SPADI), which remain susceptible to participant expectation despite blinded assessors administering and scoring the instruments. (5) Bias in selection of the reported result was low risk, as all outcomes and analyses followed the prospectively registered protocol without *post-hoc* modification. On this basis, the overall risk of bias for this trial was rated “some concerns,” driven primarily by the absence of participant and therapist blinding and the lack of a sham-injection comparator which is mentioned in Table 1.

**Table 1:** Cochrane risk-of-bias (RoB 2.0) assessment for the trial.

RoB 2.0 Domain	Judgment	Rationale
1. Randomization process	Low risk	Computer-generated allocation sequence; concealment via sequentially numbered, opaque, sealed envelopes.
2. Deviations from intended interventions	Some concerns	No participant/therapist blinding and no sham injection; standardized protocols and instructions were used to minimize differential performance.
3. Missing outcome data	Low risk	Intention-to-treat analysis; minimal attrition (≤ 2 participants per group at any time point).
4. Measurement of the outcome	Low risk (objective outcomes); Some concerns (self-reported outcomes)	Assessors and statisticians blinded to allocation; however, VAS and SPADI remain susceptible to participant expectation.
5. Selection of the reported result	Low risk	Outcomes and analyses followed the prospectively registered protocol without *post-hoc* modification.
Overall risk of bias	Some concerns	Driven primarily by the absence of participant/therapist blinding and the lack of a sham-injection comparator.

### Sample size

The required sample size was determined using G*Power (version 3.1.9.7) for a parallel two-arm design, based on detecting a 20% between-group reduction in VAS pain intensity. Previous trials of corticosteroid injection and manual therapy for subacromial shoulder pain report moderate-to-large effect sizes (Cohen's d ≈ 0.6–0.9), including Rhon DI et al. (8); a conservative effect size of d = 0.70 was therefore selected. A 20% reduction on a 10-cm VAS corresponds to a decrease of approximately 1.5–2.0 points, consistent with established MCID values for shoulder pain. With 80% power (*β* = 0.20) and a two-sided *α* of 0.05, a total sample of 60 participants (30 per group) was required.

Pain intensity (VAS) was designated *a priori* as the single primary outcome for the purpose of sample size estimation; all other endpoints (pain pressure threshold, range of motion, SPADI, and radiological measures) were classified as secondary or exploratory and were not independently powered.

### Participants

Participants were enrolled from university-level volleyball teams via posted advertisements, referrals from team physicians, and notices at sports facilities. All procedures were carried out at the Physiotherapy Clinic of Prince Sattam bin Abdulaziz University, Al Kharj, Saudi Arabia. Eligible participants were male university volleyball players only, aged 18–25 years, clinically diagnosed with subacromial impingement syndrome (SIS) by an orthopedic surgeon; only males were included to ensure a homogeneous sample and minimize sex-related differences in shoulder anatomy, biomechanics, strength, and hormonal influences that could affect treatment response, with recruitment from male teams competing at a similar level. Diagnosis combined clinical history with standardized orthopedic assessment; participants were required to have shoulder symptoms for two to six months, characterized by activity-related pain during overhead actions. Clinical confirmation required positive results on at least two of the Neer impingement test, Hawkins–Kennedy test, and painful arc test (13), plus reduced subacromial space width (≤10 mm) on high-resolution diagnostic ultrasound, performed by an experienced musculoskeletal radiologist using a standardized protocol with the shoulder in neutral alignment (14).

**Figure 1:**
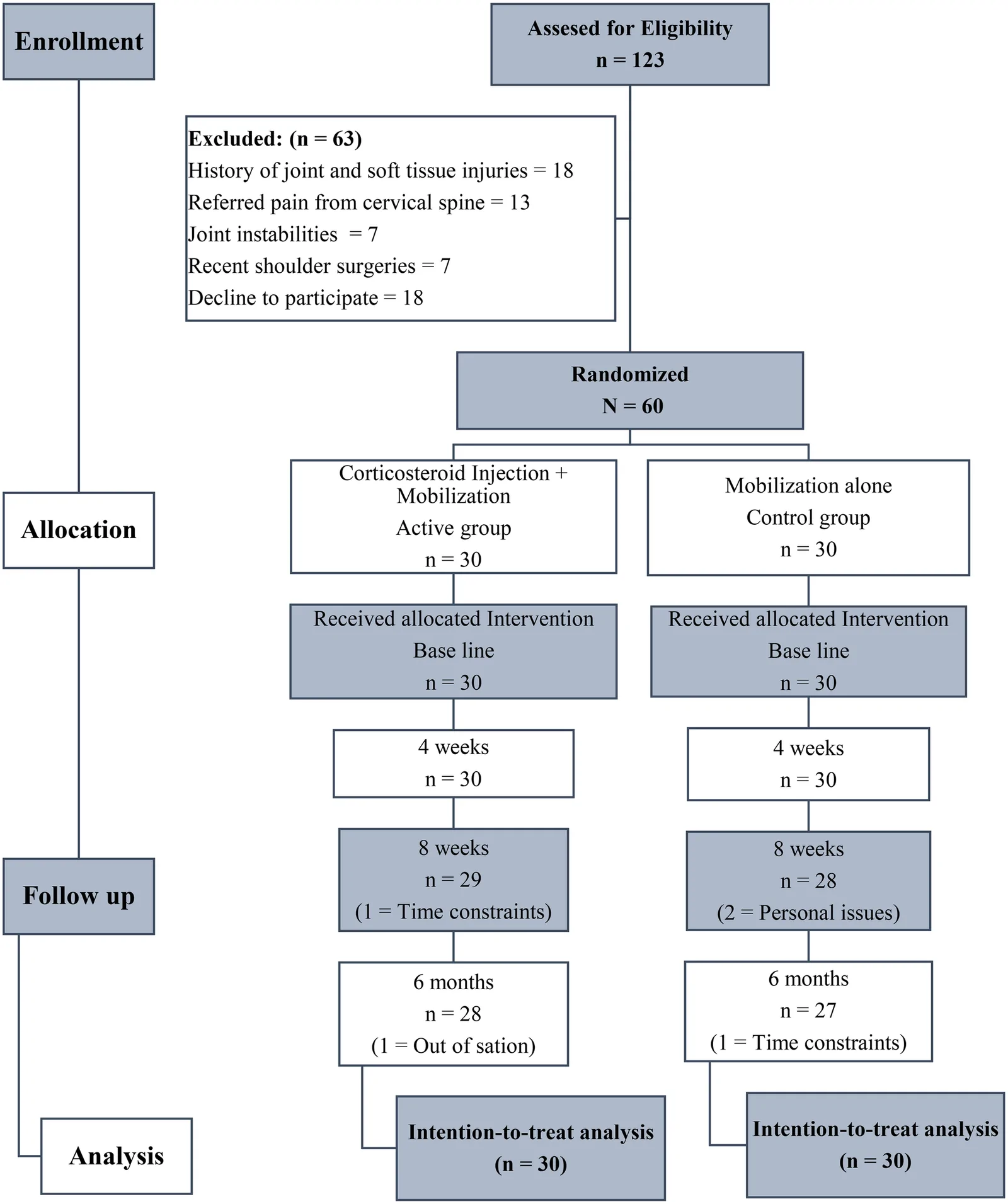
CONSORT flow diagram of participant progress through the trial.

Exclusion criteria encompassed prior shoulder injury, full-thickness rotator cuff tears, significant labral or capsular lesions, shoulder instability, fractures or dislocations, moderate-to-severe glenohumeral or acromioclavicular osteoarthritis, systemic inflammatory conditions, upper limb neurological impairments, radiating or severe shoulder/cervical pain, skin infections, and previous shoulder surgery. Participants who had received a corticosteroid injection within the preceding six months were excluded, since corticosteroids may exert lasting effects on tendon matrix composition, collagen organization, and local inflammatory mediators beyond the typical short-term analgesic window; this six-month washout reduced potential carryover effects and ensured a relatively treatment-naïve cohort. Participant recruitment, allocation, follow-up, and analysis are illustrated in the study flow diagram (Figure 1).

### Interventions

Participants who met the eligibility criteria and provided written informed consent were randomly allocated into two intervention groups using a computer-generated randomization sequence with concealed allocation. All interventions were delivered by licensed physiotherapists with a minimum of five years of clinical experience in sports musculoskeletal rehabilitation. The intervention period lasted 4 weeks, with follow-up assessments conducted at baseline, 4 weeks (end of intervention), 8 weeks, and 6 months’ post-intervention.

### Group A: corticosteroid injection combined with mobilization

Participants in Group A received a single subacromial corticosteroid injection followed by a standardized shoulder mobilization program.

### Corticosteroid injection procedure

A single dose of 80 mg methylprednisolone acetate mixed with 5 mL of 1% lidocaine was administered into the subacromial space under strict aseptic conditions, via a standardized posterolateral approach performed by a senior orthopedic physician with substantial experience in shoulder injection technique. Anatomical landmarks, including the posterolateral border of the acromion, were identified prior to needle insertion, and a predefined standardized protocol governed participant positioning, needle orientation, insertion depth, and injection rate. Correct needle placement was clinically inferred from minimal injection resistance, absence of soft-tissue back pressure, and the expected anesthetic response. No ultrasound guidance was used, consistent with routine clinical practice and with the methodology used in several comparable pragmatic trials of subacromial corticosteroid injection (15, 16). Landmark-guided subacromial injection by experienced clinicians achieves accurate bursal placement in approximately 70%–90% of cases versus over 90%–95% for ultrasound guidance, though this accuracy difference has not consistently translated into differing clinical outcomes, and placement variability remains a recognized limitation of the landmark-guided technique. Ultrasound guidance was omitted because it is not part of routine clinical practice at our institution for this indication, and because the aim was to evaluate the pragmatic, real-world effectiveness of the combined intervention as typically delivered clinically; to minimize placement variability, all injections were performed by a single senior physician following the protocol above. Participants were advised to rest the injected shoulder for 48 h and avoid overhead activities during this period (6, 15).

### Mobilization protocol

Following the 48-hour post-injection rest period, participants commenced a supervised mobilization program delivered five sessions per week for 4 weeks. The mobilization protocol was based on Maitland's concept and included graded oscillatory mobilizations targeting the glenohumeral and scapulothoracic joints. Techniques included:

- Glenohumeral joint anterior, posterior, and inferior glides (Grades I–IV)
- Scapulothoracic mobilization focusing on upward rotation and posterior tilting
- Mobilization intensity was progressed based on pain tolerance and symptom response

Each session lasted approximately 30 min and concluded with gentle active-assisted range-of-motion exercises within pain-free limits. No strengthening exercises were introduced, for two reasons. First, isolating the effects of mobilization and injection allowed observed changes in pain, mobility, and structural parameters to be attributed specifically to these interventions without the confounding influence of exercise-induced adaptations, which typically require several weeks to manifest. Second, participants presented with acute, pain-dominant SIS (symptom duration 6 weeks to 6 months) rather than chronic, strength-deficit-dominant presentations; introducing resisted loading into an inflamed, mechanically compressed subacromial space during this early phase risked provoking symptom flare and undermining adherence. Consistent with staged rehabilitation frameworks for painful shoulder conditions, pain modulation and restoration of pain-free mobility were therefore prioritized as a necessary first phase, with structured strengthening intended to follow but outside the scope of the present 4-week trial (9, 16).

### Group B: mobilization alone

Participants allocated to Group B received the same standardized mobilization protocol as described for Group A but without corticosteroid injection. The frequency, duration, and progression criteria of mobilization techniques were identical to ensure comparability between groups. Sessions were conducted five times per week for 4 weeks, with each session lasting approximately 30 min. Emphasis was placed on restoring pain-free shoulder mobility and optimizing scapulohumeral rhythm. Participants were similarly instructed to avoid aggravating overhead volleyball activities during the treatment period.

### Compliance and adverse events monitoring

Attendance was recorded to monitor compliance; participants were required to attend at least 80% of sessions to be considered protocol-adherent, with missed sessions and reasons documented. No additional physiotherapy, injections, or analgesics were permitted, and protocol deviations were documented and reviewed before analysis. Analysis followed the intention-to-treat principle, including all randomized participants regardless of adherence; missing follow-up data were handled using last observation carried forward (LOCF). No participants were excluded for non-compliance, and no cross-over occurred. LOCF was selected *a priori* given the low anticipated attrition in this closely supervised cohort; attrition was minimal in practice (maximum two participants per group at any follow-up, ≤6.7% of each arm), so its influence on results is expected to be small. Nonetheless, LOCF assumes no further change after the last observation, which can bias treatment-effect estimates in either direction, and is now generally considered inferior to likelihood-based approaches such as mixed-effects models for repeated measures (MMRM), which use all available data under a less restrictive missing-at-random assumption.

### Outcomes

All clinical outcome measures (VAS, pain pressure threshold, range of motion, SPADI) were assessed by a single independent physiotherapist-assessor, uninvolved in either intervention and not a study author, blinded to group allocation at every time point. All radiological images (MRI and ultrasound) were evaluated by the same experienced musculoskeletal radiologist who performed baseline screening, uninvolved in treatment delivery, blinded to group allocation and timepoint, and reading images in randomized, de-identified order to minimize expectation bias.

### Primary outcome

Pain intensity was assessed using a 10-cm Visual Analog Scale (VAS), a reliable (ICC 0.97; 95% CI 0.96–0.98) and validated tool for measuring subjective pain. Participants marked their current shoulder pain on a 10-cm line ranging from “0 = no pain” to “10 = worst imaginable pain,” following standardized, non-prompted instructions. The assessor then measured the mark using a calibrated transparent ruler, with higher scores indicating greater pain intensity (17).

### Key secondary outcomes

Pain pressure threshold (PPT) over the deltoid muscle was measured using a calibrated handheld digital algometer (Somedic, Hörby, Sweden) with a 1-cm^2^ rubber-tipped probe. With participants seated and the arm relaxed, pressure was applied perpendicularly at the midpoint of the lateral deltoid at 1 kg/s; participants indicated when pressure first became painful, and the mean of three readings (30–60 s apart) was used, all by the same assessor using standardized instructions. Algometry shows high intra-rater (ICC 0.85–0.97) and good inter-rater (ICC 0.75–0.90) reliability and strong validity for mechanical pain sensitivity (18).

Shoulder range of motion (abduction and external rotation) was measured using a standard universal goniometer (National 360 Goniometer, National Tools Ltd., New Delhi; ICC 0.94–0.99). For abduction, the fulcrum was placed over the anterior acromion, the stationary arm aligned with the sternum, and the moving arm following the humeral midline. For external rotation, the fulcrum was positioned over the olecranon with the arm at 90° abduction, and the arms aligned with the ulna. This method is widely recognized for reliability and validity in shoulder mobility assessment (19).

Functional disability was assessed using the Shoulder Pain and Disability Index (SPADI), a validated 13-item self-reported questionnaire measuring pain and difficulty in daily activities, rated 0–10 per item, with subscale and total scores expressed as percentages (higher = greater pain/disability). SPADI shows high internal consistency (Cronbach's alpha 0.81–0.92), excellent test–retest reliability (ICC 0.89–0.92), and good validity (20).

### Exploratory outcome

Radiographic assessments measured the coracohumeral interval, supraspinatus tendon thickness, and subacromial bursa thickness, using MRI (Koninklijke Philips N.V., Netherlands) and high-resolution ultrasound (Philips Healthcare, Netherlands), recorded in millimeters. MRI used a 1.5 T scanner with standard axial, coronal, and sagittal sequences to evaluate coracohumeral interval space and supraspinatus tendon thickness; ultrasound used a 7–15 MHz linear transducer for dynamic assessment of bursal thickness. These are validated, reliable tools for detecting rotator cuff and subacromial pathologies, with good intra- and inter-rater reliability (ICC 0.85–0.95) (21).

All outcome assessments were conducted at baseline, 4 weeks, 8 weeks, and 6 months, selected *a priori* to align imaging with clinical follow-up for concurrent paired comparison, and to capture the early post-injection window (4 weeks), short-term post-intervention window (8 weeks), and longer-term window (6 months) over which tendon and bursal remodeling might be detected. All measurements were performed by assessors blinded to group allocation.

### Statistical methods

Data were analyzed according to the intention-to-treat principle. Normality was assessed using the Shapiro–Wilk test. For normally distributed variables, a two-way repeated-measures ANOVA evaluated group (active vs. control), time (baseline, 4, 8 weeks, 6 months), and group  ×  time interaction effects; this was the primary inferential analysis. Where interaction effects were significant, *post-hoc* pairwise comparisons used Bonferroni adjustment within each outcome to control family-wise type I error across time points. For non-normally distributed variables, equivalent non-parametric tests (Mann–Whitney U and Wilcoxon signed-rank) were applied. Primary and secondary outcomes were predefined to reduce multiplicity bias; because sample size was calculated for VAS only, secondary and exploratory outcomes were not independently powered, and no formal adjustment was made for multiplicity across outcomes, so their results should be interpreted as hypothesis-generating. Effect sizes (Cohen's d and partial *η*^2^) and 95% CIs were reported alongside *p*-values, using Cohen's guidelines (0.2 = small, 0.5 = medium, 0.8 = large). All analyses used IBM SPSS Statistics (Version 30.0.0, IBM Corp., Armonk, NY, USA).

## Results

At the beginning of the study, one of the research team member screened 123 participants based on the selection criteria for inclusion in the study. Out of these, eighteen participants had a history of joint and soft tissue injuries, thirteen had referred pain from cervical spine, seven had joint instabilities and seven recently undergone joint surgeries. In addition, eighteen participants refused to participate in the study due to some personal reasons. Finally, sixty (*N* = 60) participants were recruited through random allocation method and assigned to active and control groups. The study followed the intention-to-treat principle method, in which all participant's data are included in the analysis. At the end of the 8-week period, one participant in active group and two participants in control group did not complete the follow-up measurements due to time constraints and personal reasons. Similarly, at the 6-month measurement, one participant in active group and one participant in control group did not provide measurements due to personal reasons and time constraints.

Table 2 summarizes the baseline demographic, anthropometric, sports participation, and shoulder-related characteristics of participants in the active (*n* = 30) and control (*n* = 30) groups. Independent samples *t*-tests and chi-square tests revealed no significant between-group differences at baseline (*p* > 0.05). Participants in the active and control groups were similar in age (21.11 ± 1.4 vs. 21.53 ± 1.3 years; *p* = 0.233). Anthropometric measures, including height (1.72 ± 0.16 vs. 1.71 ± 0.17 m; *p* = 0.815) and body weight (77.9 ± 7.3 vs. 78.2 ± 7.5 kg; *p* = 0.875), did not differ significantly between groups. Volleyball-specific characteristics were also comparable. Years of competitive participation (5.1 ± 1.2 vs. 5.0 ± 1.3 years; *p* = 0.742) and weekly training volume (9.6 ± 1.8 vs. 9.4 ± 1.9 h/week; *p* = 0.681) showed no significant differences. The distribution of playing positions (spiker, setter, libero, and blocker) was similar across groups (*χ*^2^, *p* > 0.05). The proportion of participants with dominant shoulder involvement was comparable between the active (70%) and control (73%) groups, with no significant difference in shoulder dominance distribution (*χ*^2^, *p* > 0.05). Overall, the two groups were well matched at baseline, supporting the internal validity of subsequent between-group comparisons.

**Table 2:** Sports participation and shoulder-specific baseline characteristics of active and control groups.

Variable	Active group (*n* = 30) Mean ± SD	Control group (*n* = 30) Mean ± SD	*p*-value
Age (y)	21.11 ± 1.4	21.53 ± 1.3	0.233
Height (meter)	1.72 ± 0.16	1.71 ± 0.17	0.815
Weight (kg)	77.9 ± 7.3	78.2 ± 7.5	0.875
Years of competitive volleyball participation (y)	5.1 ± 1.2	5.0 ± 1.3	0.742
Weekly training volume (hours/week)	9.6 ± 1.8	9.4 ± 1.9	0.681
Playing position (%)			
Spiker (Number/Percentage)	14 (47%)	13 (43%)	–
Setter (Number/Percentage)	6 (20%)	7 (23%)	–
Libero (Number/Percentage)	5 (17%)	5 (17%)	–
Blocker (Number/Percentage)	5 (17%)	5 (17%)	–
Dominant shoulder affected N (%)	21 (70%)	22 (73%)	–
Non-dominant shoulder affected N (%)	9 (30%)	8 (27%)	–

SD, standard deviation; y, year; kg, kilogram.

### Primary outcome

#### Pain intensity

A significant group  ×  time interaction was observed for pain intensity (*p* = 0.001), indicating differential treatment responses over time. Although both groups showed reductions in pain, the active group demonstrated faster and substantially greater improvement. Between-group differences favored the active group at 4 weeks (MD = 2.0; 95% CI: 1.62–2.38), 8 weeks (MD = 2.7; 95% CI: 2.35–3.05), and 6 months (MD = 2.6; 95% CI: 2.31–2.88). These differences corresponded to moderate standardized effects at 4 weeks (Cohen's d ≈ 0.8) and large effects at 8 weeks and 6 months (d ≈ 1.2–1.4), with a large group  ×  time effect (partial *η*^2^ ≈ 0.20–0.30). Clinically, this pattern suggests that the addition of corticosteroid injection provides earlier pain modulation, facilitating sustained symptom relief beyond the short term.

In the context of competitive volleyball players, a between-group difference of 2.6 points on a 10-point VAS scale represents a clinically meaningful reduction in pain during high-demand overhead activities. Established MCID values for shoulder pain range between 1.4 and 2.0 points; therefore, the observed differences not only achieved statistical significance but exceeded thresholds considered meaningful for patient-perceived improvement. Furthermore, the large standardized effect sizes observed at 8 weeks and 6 months (Cohen's d > 1.0) indicate substantial treatment impact, suggesting that the combined intervention may meaningfully influence return-to-sport readiness and training tolerance in young athletes.

### Key secondary outcomes

#### Pain pressure threshold

Pain pressure threshold increased significantly over time in the active group, while early changes in the control group were small and non-significant. By 6 months, the between-group difference was significant (MD = −25.2 kPa; 95% CI: −41.8 to −8.6; *p* = 0.001), representing a moderate effect size (d ≈ 0.6–0.7) and a moderate interaction effect (partial *η*^2^ ≈ 0.10–0.15). This finding indicates enhanced peripheral pain tolerance in the active group, suggesting improved nociceptive modulation when corticosteroid injection is combined with mobilization.

#### Shoulder range of motion

Both shoulder abduction and external rotation improved over time in both groups; however, improvements were consistently greater in the active group (group  ×  time interaction, *p* = 0.001). For shoulder abduction, the between-group difference increased progressively, reaching −14.6° (95% CI: −17.6 to −11.5) at 6 months. This represents a large standardized effect (d ≈ 1.3–1.5) and a large interaction effect (partial *η*^2^ > 0.20), indicating clinically meaningful restoration of overhead shoulder function.

For external rotation, between-group differences were smaller at early follow-ups but reached −6.4° (95% CI: −8.1 to −4.7) at 6 months, corresponding to a moderate-to-large effect (d ≈ 0.8–1.0). These findings suggest that early pain reduction may enable more effective gains in shoulder mobility over time. Clinically, an additional 14.6° improvement in abduction at 6 months may represent meaningful restoration of overhead reach capacity, which is essential for volleyball-specific performance tasks. The large effect sizes (d ≈ 1.4–1.6) observed for SPADI at 6 months indicate substantial functional recovery. In high-demand athletic populations, such magnitude of improvement may correspond to enhanced performance capacity, reduced compensatory movement patterns, and improved confidence during overhead sport-specific tasks.

#### Functional disability (SPADI)

Functional disability improved significantly in both groups; however, the magnitude of improvement was substantially greater in the active group across all follow-ups. At 6 months, the between-group difference was 10.7 points (95% CI: 9.3–12.1; *p* = 0.001), exceeding the minimal clinically important difference for SPADI. This difference corresponds to a large effect size (d ≈ 1.4–1.6) and a large group  ×  time effect (partial *η*^2^ ≈ 0.25–0.35), indicating that the combined intervention resulted in superior and sustained functional recovery. These findings are particularly relevant for overhead athletes, where functional capacity is critical for return to sport.

### Exploratory outcome

#### Imaging outcomes

MRI assessment of the coracohumeral interval space demonstrated significant increases in both groups, with consistently greater improvements in the active group. At baseline, the coracohumeral interval measured 5.9 ± 0.6 mm in the active group and 5.8 ± 0.6 mm in the control group. At 6 months, values increased to 8.6 ± 0.7 mm and 6.6 ± 0.6 mm, respectively. The between-group difference at 6 months (−2.0 mm; 95% CI: −2.4 to −1.6; *p* = 0.001) represents a large effect size (d ≈ 1.2–1.4) and suggests meaningful structural decompression of the subacromial region. Ultrasound measures showed progressive reductions in supraspinatus tendon thickness and subacromial bursa thickness in both groups. Early between-group differences were small and non-significant; however, moderate effects emerged by 8 weeks, progressing to large effects by 6 months (d ≈ 0.9–1.2; *p* = 0.001). This temporal pattern indicates delayed but clinically relevant tissue adaptation associated with the combined intervention.

Collectively, these findings demonstrate that corticosteroid injection combined with mobilization produces consistently larger and clinically meaningful improvements in pain, shoulder mobility, function, and structural measures compared with mobilization alone. Standardized effect sizes indicate moderate benefits in the early phase and large, durable effects by 6 months, supporting the efficacy of a multimodal approach for managing subacromial impingement syndrome in high-demand athletic populations (Tables 3, 4).

**Table 3:** Pre- and post-intervention primary and secondary outcome measures of active and control groups.

Variable	Duration	Active group (*n* = 30) Mean ± SD	Control group (*n* = 30) Mean ± SD	Group × Time
Pain intensity—VAS (0–10 cm)	Baseline	7.6 ± 1.1	7.5 ± 1.2	*p* = 0.001^a^
	4 weeks	3.9 ± 1.0	5.9 ± 1.2	
	8 weeks	1.9 ± 0.6	4.6 ± 0.9	
	6 months	1.1 ± 0.3	3.7 ± 0.8	
Pain pressure threshold—Deltoid (kPa)	Baseline	300.8 ± 29.4	302.6 ± 30.2	*p* = 0.001^a^
	4 weeks	312.5 ± 30.6	307.2 ± 30.9	
	8 weeks	326.8 ± 31.1	313.6 ± 31.0	
	6 months	345.7 ± 31.9	320.5 ± 31.4	
Shoulder abduction (degree)	Baseline	44.5 ± 4.3	45.1 ± 4.2	*p* = 0.001^a^
	4 weeks	57.6 ± 4.9	54.1 ± 4.7	
	8 weeks	66.1 ± 5.4	60.4 ± 5.0	
	6 months	78.4 ± 6.1	63.8 ± 5.4	
Shoulder—External rotation (degree)	Baseline	27.6 ± 2.0	27.8 ± 2.1	*p* = 0.001*
	4 weeks	30.9 ± 2.5	29.7 ± 2.4	
	8 weeks	33.8 ± 2.9	30.8 ± 2.5	
	6 months	39.2 ± 3.8	32.8 ± 2.7	
Functional disability (SPADI)	Baseline	47.1 ± 4.6	46.8 ± 4.5	*p* = 0.001*
	4 weeks	34.1 ± 4.0	43.8 ± 4.6	
	8 weeks	25.6 ± 3.1	36.7 ± 3.7	
	6 months	19.2 ± 1.6	29.9 ± 3.0	
MRI—Coracohumeral interval space (mm)	Baseline	5.9 ± 0.6	5.8 ± 0.6	*p* = 0.001*
	4 weeks	6.8 ± 0.6	6.0 ± 0.6	
	8 weeks	7.6 ± 0.6	6.3 ± 0.6	
	6 months	8.6 ± 0.7	6.6 ± 0.6	
Ultrasound—Supraspinatus tendon thickness (mm)	Baseline	7.0 ± 0.7	6.9 ± 0.7	*p* = 0.001*
	4 weeks	6.6 ± 0.6	6.8 ± 0.6	
	8 weeks	6.3 ± 0.5	6.6 ± 0.6	
	6 months	5.8 ± 0.4	6.3 ± 0.5	
Ultrasound—Thickness of Subacromial bursa (mm)	Baseline	11.1 ± 0.9	11.0 ± 0.8	*p* = 0.001*
	4 weeks	10.7 ± 0.9	10.9 ± 0.8	
	8 weeks	10.3 ± 0.8	10.6 ± 0.7	
	6 months	9.9 ± 0.7	10.4 ± 0.6	

*Significant, SD, standard deviation; VAS, visual analog scale; cm, centimeter; kPa, kilopascal, SPADI, Shoulder Pain and Disability Index; MRI, magnetic resonance imaging; mm, millimeter.

**Table 4:** Mean (95% CI) difference between groups for primary and secondary outcome variables.

Variables	Difference within groups						Difference between groups		
	Week 4 minus Baseline		Week 8 minus Baseline		Month 6 minus Baseline		4 weeks	8 weeks	6 months
	Active Mean ± SD	Control Mean ± SD	Active Mean ± SD	Control Mean ± SD	Active Mean ± SD	Control Mean ± SD	Active—Control Mean ± SD	Active—Control Mean ± SD	Active—Control Mean ± SD
Pain intensity	−3.7 ± 0.6	−1.6 ± 0.7	−5.7 ± 0.6	−2.9 ± 0.8	−6.5 ± 0.6	−3.8 ± 0.7	2.0 (1.62 to 2.38)	2.7 (2.35 to 3.05)	2.6 (2.31 to 2.88)
	*p* = 0.001	*p* = 0.001	*p* = 0.001	*p* = 0.001	*p* = 0.001	*p* = 0.001	*p* = 0.001	*p* = 0.001	*p* = 0.001
Pain pressure threshold	11.7 ± 20.8	4.6 ± 21.0	26.0 ± 21.1	11.0 ± 21.4	44.9 ± 21.3	17.9 ± 21.6	−5.3 (−21.6 to 11.0)	−13.2 (−29.5 to 3.1)	−25.2 (−41.8 to −8.6)
	*p* = 0.512*	*p* = 0.931*	*p* = 0.008	*p* = 0.441*	*p* = 0.001	*p* = 0.162*	*p* = 0.521*	*p* = 0.109*	*p* = 0.001
Shoulder abduction	13.1 ± 3.6	9.0 ± 3.4	21.6 ± 3.8	15.3 ± 3.6	33.9 ± 4.1	18.7 ± 3.9	−3.5 (−5.9 to −1.1)	−5.7 (−8.3 to −3.1)	−14.6 (−17.6 to −11.5)
	*p* = 0.001	*p* = 0.001	*p* = 0.001	*p* = 0.001	*p* = 0.001	*p* = 0.001	*p* = 0.004	*p* = 0.001	*p* = 0.001
Shoulder External rotation	3.3 ± 1.7	1.9 ± 1.6	6.2 ± 1.8	3.0 ± 1.7	11.6 ± 2.0	5.0 ± 1.8	−1.2 (−2.5 to 0.1)	−3.0 (−4.4 to −1.6)	−6.4 (−8.1 to −4.7)
	*p* = 0.001	*p* = 0.002	*p* = 0.001	*p* = 0.001	*p* = 0.001	*p* = 0.001	*p* = 0.073*	*p* = 0.001	*p* = 0.001
Functional disability	−13.0 ± 2.4	−3.0 ± 2.8	−21.5 ± 2.6	−10.1 ± 2.9	−27.9 ± 2.7	−16.9 ± 3.0	9.7 (7.5 to 11.8)	11.1 (9.3 to 12.9)	10.7 (9.3 to 12.1)
	*p* = 0.001	*p* = 0.00	*p* = 0.001	*p* = 0.001	*p* = 0.001	*p* = 0.001	*p* = 0.001	*p* = 0.001	*p* = 0.001
Coracohumeral interval space	0.9 ± 0.4	0.2 ± 0.4	1.7 ± 0.4	0.5 ± 0.4	2.7 ± 0.5	0.8 ± 0.4	−0.8 (−1.1 to −0.5)	−1.3 (−1.6 to −0.9)	−2.0 (−2.4 to −1.6)
	*p* = 0.001	*p* = 0.618*	*p* = 0.001	*p* = 0.013	*p* = 0.001	*p* = 0.001	*p* = 0.001	*p* = 0.001	*p* = 0.001
Supraspinatus tendon thickness	−0.4 ± 0.3	−0.1 ± 0.4	−0.7 ± 0.3	−0.3 ± 0.4	−1.2 ± 0.3	−0.6 ± 0.4	0.2 (−0.1 to 0.5)	0.3 (0.1 to 0.6)	0.5 (0.3 to 0.8)
	*p* = 0.041	*p* = 0.904*	*p* = 0.001	*p* = 0.182*	*p* = 0.001	*p* = 0.001	*p* = 0.226*	*p* = 0.001	*p* = 0.001
Thickness of Subacromial bursa	−0.4 ± 0.5	−0.1 ± 0.5	−0.8 ± 0.5	−0.4 ± 0.5	−1.2 ± 0.5	−0.6 ± 0.5	0.2 (−0.3 to 0.6)	0.3 (−0.1 to 0.7)	0.5 (0.1 to 0.9)
	*p* = 0.281*	*p* = 0.691*	*p* = 0.011	*p* = 0.118*	*p* = 0.001	*p* = 0.006	*p* = 0.442*	*p* = 0.124*	*p* = 0.001

*Non-significant.

Although statistically significant between-group differences were observed in radiological parameters, imaging findings were interpreted in conjunction with clinical outcomes. Structural changes in the subacromial region may not directly correspond to symptomatic improvement, and radiological adaptations are often slower to manifest compared with pain and functional recovery. Therefore, imaging improvements in the present study should be considered supportive of improved subacromial mechanics rather than definitive indicators of symptom resolution.

##### Adverse events

No serious adverse events were observed in either group during the intervention or follow-up periods. In the corticosteroid injection group, two participants reported mild, transient post-injection soreness lasting less than 48 h, which resolved without additional treatment. No infections, allergic reactions, neurovascular complications, or persistent symptom exacerbations were reported. No adverse events were recorded in the mobilization-only group. No participants discontinued the study due to treatment-related complications.

## Discussion

This prospective randomized controlled trial investigated the comparative effects of corticosteroid injection combined with mobilization versus mobilization alone on pain, function, shoulder mobility, and radiological outcomes in university volleyball players with subacromial impingement syndrome (SIS). The principal findings indicate that the combined intervention resulted in earlier, greater, and more sustained improvements across all clinical and imaging outcomes, with moderate to large standardized effect sizes and changes that met or exceeded established minimum clinically important difference (MCID) thresholds for key outcomes.

Pain reduction, measured using the visual analog scale (VAS), was significantly greater in the combined intervention group at all follow-up points. The between-group differences (2.0–2.7 points on a 0–10 scale) exceeded the commonly accepted MCID for shoulder pain, which ranges from 1.4 to 2.0 points depending on baseline severity and population characteristics (22, 23). These findings indicate that the observed reductions were not merely statistically significant but also clinically meaningful, particularly for overhead athletes who require pain-free shoulder function for optimal performance. The large effect sizes observed at 8 weeks and 6 months suggest that corticosteroid injection provides effective early pain modulation, which may facilitate engagement in mobilization and therapeutic exercises. This supports prior evidence demonstrating that corticosteroid injections offer superior short-term pain relief compared with exercise alone in SIS, although their long-term benefits remain debated (24). In the present study, the persistence of pain reduction at 6 months suggests a synergistic benefit when injections are combined with structured mobilization.

Functional disability, assessed using the Shoulder Pain and Disability Index (SPADI), improved significantly in both groups; however, the combined intervention produced substantially greater reductions. The between-group difference at 6 months (10.7 points) was statistically significant and accompanied by large effect sizes. Reported MCID values for SPADI in shoulder disorders range from 8 to 13 points, while the minimal detectable change (MDC) has been reported between 18 and 21 points (25, 26). Although the between-group difference approached the lower end of MCID estimates, the within-group improvement in the active group exceeded MDC values, indicating true change beyond measurement error. For athletic populations such as volleyball players, even modest improvements in SPADI scores may represent meaningful gains in sport-specific tasks, training tolerance, and return-to-play readiness. These findings are consistent with previous trials demonstrating greater functional recovery when pain reduction enables more effective rehabilitation participation (7).

Improvements in shoulder abduction and external rotation were significantly greater in the combined intervention group, with large effect sizes observed for abduction and moderate-to-large effects for external rotation. Loss of range of motion (ROM) is a defining characteristic of SIS and has been strongly associated with impaired overhead athletic performance (27). The greater ROM gains observed in the active group likely reflect reduced pain inhibition and improved capsulomechanical properties following mobilization. These results align with previous studies reporting enhanced ROM when manual therapy is combined with interventions that reduce inflammatory pain (28). Importantly, the progressive increase in between-group differences over time suggests that early pain relief may enable cumulative mechanical and neuromuscular adaptations during rehabilitation.

Radiological findings demonstrated greater increases in coracohumeral interval space and greater reductions in supraspinatus tendon and subacromial bursa thickness in the combined intervention group. However, caution is warranted when interpreting these structural changes. The relationship between imaging findings and symptom severity in subacromial impingement syndrome remains complex and sometimes inconsistent. Structural abnormalities may be present in asymptomatic individuals, and imaging improvements do not always directly correspond to pain resolution (29, 30). Furthermore, structural adaptations typically evolve more gradually than symptomatic improvement. Pain reduction may occur rapidly following anti-inflammatory intervention, whereas measurable changes in tendon morphology or bursal thickness may require prolonged biomechanical normalization. Therefore, the observed radiological improvements at 6 months likely reflect cumulative mechanical and inflammatory modulation rather than an immediate structural “reversal” of pathology. These radiological changes should be interpreted as supportive evidence of improved subacromial mechanics rather than definitive proof of structural recovery. Because both study arms received an identical mobilization program and differed only in the addition of the corticosteroid injection, the greater increase in coracohumeral interval observed in the combined group cannot be attributed to mobilization alone and is more plausibly linked, at least in part, to the injection. We do not believe the corticosteroid itself directly enlarges the bony coracohumeral distance; rather, the coracohumeral interval is a composite, position-dependent measure that is reduced not only by bone geometry but by the volume of soft tissue occupying the subacromial space (bursal effusion and supraspinatus tendon thickness) and by the resting position of the humeral head. A plausible explanation is that corticosteroid-mediated reduction of local inflammatory bursal effusion and tendon thickness—changes we also observed on ultrasound in the same group—mechanically freed up measurable subacromial space, while more effective, less pain-inhibited rotator cuff and scapular muscle activation during mobilization may have reduced superior humeral head migration. Both mechanisms would be expected to act in the same direction on this measure without requiring any direct structural “regrowth” of bone or soft tissue. We have therefore avoided attributing the coracohumeral interval change to a direct anti-inflammatory or trophic effect of the injection on periarticular tissue, and instead attribute it to this combination of reduced peri-tendinous swelling and improved dynamic humeral head positioning; we agree this remains an inferential interpretation that was not directly tested (e.g., via correlation with humeral head migration or muscle activation measures) and should be treated as hypothesis-generating. Nonetheless, the magnitude and consistency of changes in the combined group, together with sustained clinical improvement, suggest a plausible interaction between mechanical decompression and symptom reduction.

Previous randomized trials indicate that corticosteroid injections provide superior short-term pain relief compared with exercise therapy alone; however, their long-term benefits appear to diminish beyond 3–6 months (24, 31). Although injections effectively reduce inflammation, they do not correct underlying biomechanical impairments such as capsular tightness, altered scapular kinematics, or rotator cuff dysfunction. In contrast, exercise-based rehabilitation and supervised physical therapy consistently improve pain and function in SIS and are widely regarded as first-line treatments (7, 28). Nevertheless, in pain-dominant cases—particularly among overhead athletes—symptom severity may limit early rehabilitation participation, and mechanical correction alone may not fully address pain-related neuromuscular inhibition. Comparative studies suggest that manual therapy and corticosteroid injection yield similar one-year outcomes, indicating that each targets distinct aspects of SIS pathology (8).

The beneficial effects of the combined intervention likely reflect complementary mechanisms. Corticosteroid injections exert potent anti-inflammatory effects by suppressing pro-inflammatory cytokines, inhibiting phospholipase A2 activity, and reducing the production of prostaglandins and other nociceptive mediators within the subacromial space, thereby decreasing peripheral sensitization and pain signaling at both peripheral and central levels (24). It should be noted that, as injections were landmark-guided rather than ultrasound-verified, the precision of bursal drug delivery could not be confirmed for individual participants, and the anti-inflammatory effect described here should be interpreted as an average effect across the active group rather than a uniformly precise dose delivered to every participant. By reducing inflammatory pain, corticosteroid injection may restore more normal neuromuscular activation, creating a favorable physiological environment for rehabilitation. Mobilization techniques may restore joint mechanics, reduce capsular stiffness, and improve proprioceptive input, thereby enhancing motor control and load tolerance. Mechanically, mobilization improves capsular extensibility, reduces adhesions, enhances joint lubrication, and restores accessory joint glides necessary for pain-free elevation and rotation (9). Neurophysiologically, joint mobilization stimulates type I and II mechanoreceptors, modulates dorsal horn activity, and activates descending inhibitory pathways, contributing to additional analgesic effects beyond mechanical correction alone (25). The synergistic interaction between these interventions may follow a sequential facilitation model: early pharmacological reduction of inflammation and nociception permits more effective application of mobilization techniques, which subsequently restore joint mechanics and neuromuscular control. In young athletic populations, greater tissue adaptability and neuromuscular plasticity may amplify this interaction, allowing restoration of optimal movement patterns before maladaptive compensations develop (27). While corticosteroid injection alone may provide short-term symptom relief and mobilization alone may be limited by pain inhibition, their combination leverages early anti-inflammatory analgesia to enable effective mechanical restoration, resulting in durable improvements in pain, function, and structural parameters.

### Limitations

This study has several limitations. First, follow-up was limited to 6 months, so long-term durability and recurrence rates remain unknown. Return-to-sport exposure and training intensity were not systematically recorded, and adherence to activity restrictions outside the clinic could not be objectively verified. Second, participant blinding was not feasible due to the absence of a sham injection, introducing possible expectancy effects; a formal Cochrane RoB 2.0 assessment rated deviations from intended interventions and outcome measurement as “some concerns,” with an overall trial-level judgment of “some concerns.” Only male athletes were included, limiting generalizability to female players. Third, missing data were handled using last observation carried forward (LOCF); although attrition was minimal (≤2 participants per group), LOCF is conservative and can bias effect estimates, so future trials should preferentially use mixed-effects models for repeated measures (MMRM) or multiple imputation. Fourth, corticosteroid injections used anatomical landmarks without ultrasound guidance; although a single experienced physician followed a standardized technique, needle placement accuracy could not be radiologically confirmed, and some injections may have been partially extra-bursal—likely attenuating rather than inflating the observed effect. Fifth, strengthening exercises were intentionally excluded during the 4-week intervention to isolate mobilization and injection effects and avoid provoking symptoms, limiting applicability to comprehensive rehabilitation programs and potentially affecting durability and recurrence risk. Sixth, correlation analyses between imaging changes and patient-reported outcomes were not conducted, so the link between structural and functional recovery remains uncertain. Repeated reliability testing of radiological measures at follow-up was not performed. Finally, as the trial was powered solely for the primary VAS outcome, secondary and exploratory findings warrant cautious interpretation regarding multiplicity.

### Future recommendations

Future studies should include both male and female athletes and consider sex-stratified analyses. Future studies should incorporate a subsequent progressive strengthening phase (e.g., beginning once pain and range of motion have stabilized) to evaluate whether the early gains from combined corticosteroid injection and mobilization are maintained, amplified, or diminished when integrated into a complete, real-world rehabilitation pathway, and should assess strength, scapular kinematics, and recurrence alongside pain and functional outcomes. Trials should also include standardized return-to-sport criteria, performance-based measures, and longer follow-up periods to evaluate recurrence and sustained athletic participation. Additionally, establishing athlete-specific MCID thresholds and further exploring the relationship between structural imaging changes and clinical outcomes would improve clinical applicability.

### Clinical implications

From a clinical perspective, the findings suggest that corticosteroid injection should not be viewed as a stand-alone treatment, but rather as an adjunct to active rehabilitation. In university-level volleyball players with SIS, combining injection with mobilization appears to optimize pain relief and facilitate functional recovery; the accompanying radiological changes are best viewed as exploratory, hypothesis-generating findings rather than confirmed structural benefits.

## Conclusion

In conclusion, corticosteroid injection combined with mobilization resulted in greater and clinically meaningful improvements in pain, shoulder function, range of motion, and radiological parameters compared with mobilization alone in university volleyball players with subacromial impingement syndrome. As correlation analyses between imaging changes and patient-reported outcomes were not performed, these radiological findings should be interpreted as exploratory and hypothesis-generating rather than as established markers of structural recovery. However, the findings are limited to young male athletes and may not generalize to other populations. As only a single injection was studied, the long-term effects of repeated corticosteroid use remain uncertain, particularly given potential risks to tendon and cartilage tissue. Therefore, corticosteroid injections should be used cautiously as an adjunct to structured rehabilitation rather than as a standalone treatment. Overall, a multimodal approach appears beneficial, but careful clinical application and further long-term research are warranted.

## Data Availability

The raw data supporting the conclusions of this article will be made available by the authors, without undue reservation.
